# Are researchers in academic medicine flourishing? A survey of midcareer Ph.D. and physician investigators

**DOI:** 10.1017/cts.2023.525

**Published:** 2023-04-17

**Authors:** Linda H. Pololi, Arthur T. Evans, Janet T. Civian, Lisa A. Cooper, Brian K. Gibbs, Kacy Ninteau, Rada K. Dagher, Kimberly Bloom-Feshbach, Robert T. Brennan

**Affiliations:** 1 Brandeis University, Waltham, Massachusetts, USA; 2 Weill Cornell Medical College, New York, USA; 3 John Hopkins University School of Medicine and Bloomberg School of Public Health, Baltimore, Maryland, USA; 4 UMass Memorial Health Care, Worcester, Massachusetts, USA; 5 National Institute on Minority Health Disparities (Division of Clinical and Health Services Research), National Institutes of health, USA

**Keywords:** Physician investigators, faculty, academic medicine, diversity, gender, mentoring, research faculty, translational research, retention, culture, relationships, burnout, self-efficacy, career advancement, PhD, race, ethnicity

## Abstract

**Introduction:**

Midcareer research faculty are a vital part of the advancement of science in U.S. medical schools, but there are troubling trends in recruitment, retention, and burnout rates.

**Methods:**

The primary sampling frame for this online survey was recipients of a single R01 or equivalent and/or K-award from 2013 to 2019. Inclusion criteria were 3–14 years at a U.S. medical school and rank of associate professor or two or more years as assistant professor. Forty physician investigators and Ph.D. scientists volunteered for a faculty development program, and 106 were propensity-matched controls. Survey items covered self-efficacy in career, research, work-life; vitality/burnout; relationships, inclusion, trust; diversity; and intention to leave academic medicine.

**Results:**

The majority (52%) reported receiving poor mentoring; 40% experienced high burnout and 41% low vitality, which, in turn, predicted leaving intention (*P* < 0.0005). Women were more likely to report high burnout (*P* = 0.01) and low self-efficacy managing work and personal life (*P* = 0.01) and to be seriously considering leaving academic medicine than men (*P* = 0.003). Mentoring quality (*P* < 0.0005) and poor relationships, inclusion, and trust (*P* < 0.0005) predicted leaving intention. Non-underrepresented men were very likely to report low identity self-awareness (65%) and valuing differences (24%) versus underrepresented men (25% and 0%; *P* < 0.0005). Ph.D.s had lower career advancement self-efficacy than M.D.s (*P* < .0005).

**Conclusions:**

Midcareer Ph.D. and physician investigators faced significant career challenges. Experiences diverged by underrepresentation, gender, and degree. Poor quality mentoring was an issue for most. Effective mentoring could address the concerns of this vital component of the biomedical workforce.

## Introduction

Physician investigators and Ph.D. scientists are critically important for scientific discovery and its translation and application to the care of patients. Exploration and assessment of the vitality of research faculty has received scant attention, even though there are several troubling trends among research faculty in US medical schools. The average age for both physician investigators and all recipients of National Institutes of Health (NIH) research project grants has been steadily increasing (i.e., first entry into the independent research workforce) [1], and about 40% of recipients of first R01 grants do not continue their research careers with federal funding. The proportion of funded physician investigators in the biomedical research workforce has been steadily decreasing [2]. Of increasing concern are burnout rates and structural barriers that have historically limited appropriate representation by gender, race, and ethnicity.

Reasons for some of these untoward trends include difficulty in obtaining research funding; balancing clinical, research, and education responsibilities; loan repayment; discriminatory behaviors and experiences; integrating work and personal roles; and insufficient mentoring [3,4]. Recently, studies have highlighted the stress and differential impact of the SARS-CoV-2 pandemic on underrepresented physician investigators [5] and on female faculty [6].

Multiple studies have found burnout rates among medical faculty above 40% [7,12]. Whereas burnout’s causes and effects in physicians generally have been studied, there has been less focus on burnout among physician investigators and Ph.D. scientists. High burnout rates have been reported among early career clinical investigators. A 2014 study of physician scientists who received new K08 and K23 awards from NIH between 2006 and 2009 reported 41% burnout among women and 32% among men [14,15].

The NIH have reported that in fiscal year 2021, three quarters of awards were given to White investigators and one in five to Asian investigators [1]. There has been little growth in Black/African American (3% of principal investigators) and Native American awardees (0.02% of principal investigators) [1]. Women received 34% of NIH R01 awards in FY 2021 [1], and 34% of CTSA Program principal investigators in FY 2020 were women [15]. Since its 2014 report [2], the NIH have sought to address the decreased numbers of physician investigators [16], and the disproportionately low numbers of women and members of underrepresented racial and ethnic groups among awardees [17–21]. For example, in 2022, the NIH UNITE initiative launched a new program to award up to $20 million per year to underrepresented scientists [22].

Our purpose in this study of research faculty is to identify aspects of investigator experiences in need of attention in order to sustain faculty vitality and accomplishments and assess whether these challenges differ across demographic subgroups.

## Methods

### Recruitment of Study Participants

We received NIH funding to test a mentoring intervention to improve the vitality and career advancement of midcareer researchers at US medical schools. We used the baseline measurements for that randomized controlled trial as the data for this study.

We recruited a purposefully diverse non-random sample of eligible midcareer researchers to participate in the mentoring intervention. Inclusion criteria were (1) appointment for 3–14 years at a US medical school or teaching hospital; (2) rank of assistant professor (for at least 2 years) or associate professor; and (3) demonstrated research success, defined as current or recent first-time NIH R01 or R01-equivalent award; R21 or R34 award; HRSA, ARHQ or other federal agency major grant; K training grant; or recent major foundation or professional organization grant.

To obtain the sampling frame, NIH RePORTER [23] was searched for all awardees receiving qualifying grants from 2013 to 2019. Awardees who had received a prior R01 or comparable grant were omitted. Because the trial design required 50% participation by those in NIH-designated underrepresented racial and ethnic groups (Black/African American, Hispanic/Latinx, Native American, Alaska Native, or Pacific Islander) [24], additional methods were used to recruit this population, including contacting deans and others with responsibility for diversity at medical schools to enlist their help.

**Figure 1.**
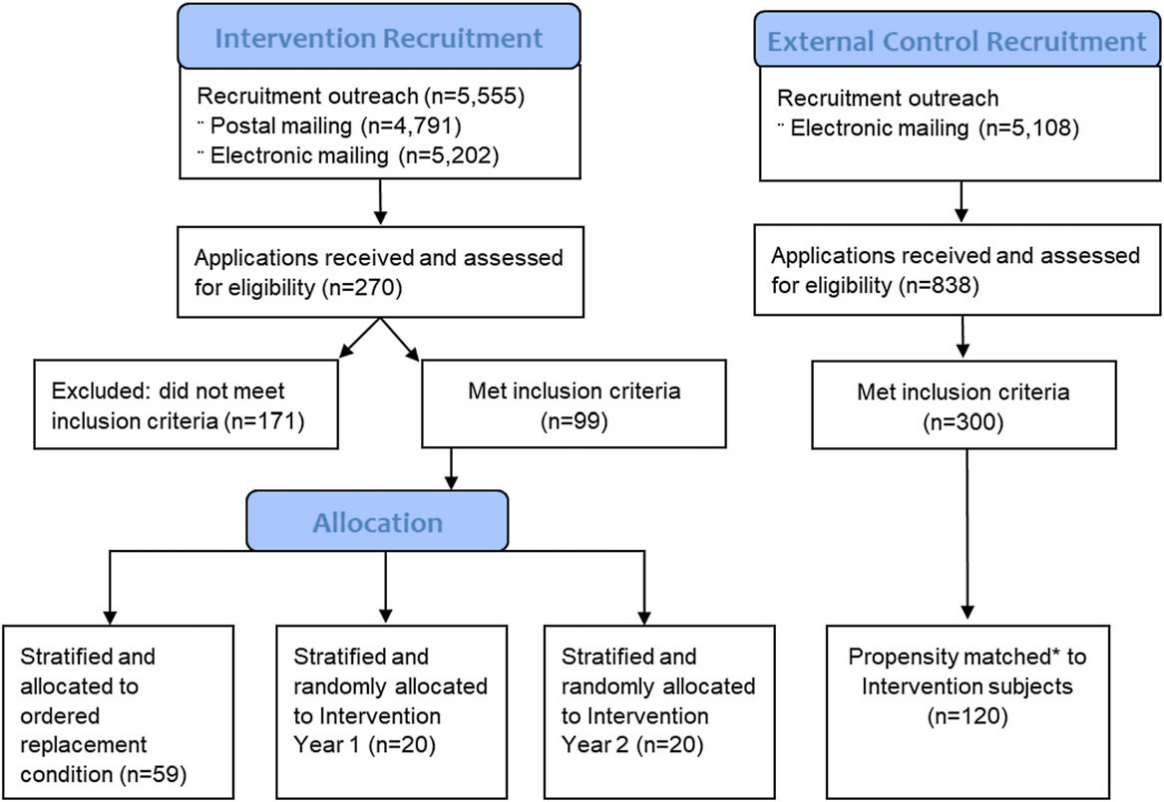
**Recruitment and allocation of study subjects.** * matching was done using an optimal matching propensity procedure with eight standardized variables collected from applications: gender, underrepresentation, rank, years of experience, number of publications, number of grants (weighted by type), M.D. vs Ph.D., and number of R01 or equivalent grants.

Through email and personalized letters, we invited 5555 individuals to submit applications to participate in a mentoring intervention that involved quarterly, in-person, two-day meetings. Of 270 received, 99 applicants met all inclusion criteria and 40 were offered places in the mentoring program. A stratified randomized selection process was designed to assure nearly equal numbers of men and women, in terms of race and ethnicity, roughly half underrepresented in medicine as defined by NIH and half non-underrepresented, and nearly equal numbers of Ph.D. and M.D. or M.D./Ph.D. Fig. 1 displays the recruitment and allocation process for the mentoring intervention subjects as well as for external control subjects described next.

We conducted a second recruitment – this time for external control subjects – using the database created to recruit intervention subjects. Emails were sent to 5108 researchers who were invited to participate in the study and offered financial incentives to complete surveys. We received 838 applications and selected 120 faculty who were statistically matched to the attributes of the 40 intervention subjects using an optimal matching propensity procedure using MatchIt [25] in R [26] (Fig. 1). Optimal matching, as opposed to nearest neighbor, minimizes total distances between treated and untreated subjects and their matches. While 120 applicants were matched, five failed to enroll in the study and seven were excluded from this investigation because they were deemed too senior (i.e., more than 1 R01 or rank of professor), for a sample size of 108 external controls. Including intervention and external control subjects, the sample totaled 148.

### Survey Instrument and Data Collection

Drawing on items from the widely used nationally validated C-Change Faculty Survey [9,11], we used scales to assess faculty regarding their vitality; relationships, inclusion, and trust; self-efficacy in the three domains of career advancement, research, and work-life integration; and the adequacy and quality of mentoring received by the faculty. Two additional measures assessed burnout (a single item) and intention to leave academic medicine. To understand faculty awareness, beliefs, and behaviors related to cross-cultural issues, we developed new items exploring identity self-awareness, valuing diversity with a focus on recruitment and workplace interactions, and anti-sexism and anti-racism skills. Table 1 shows the properties of these measures. Although all variables were analyzed taking full advantage of their ordinal or continuous characteristics, we simplified presentation of results by dichotomizing at a threshold that the authors/investigators considered as representing dysfunctional, problematic, inadequate, or clearly unsatisfactory conditions (Table 1, columns 3 and 4).

**Table 1.** Description, number of items, response scale, definition of individual mean scores of concern, and reliability of C-Change assessment scales

Scale description (number of items)	Response scale	Individual scale scores of concern		Estimated Cronbach’s alpha
		Cut point	Included responses	
**Mentoring quality (10)** *Helpfulness of mentoring received*	1–6 Extent	< 3.5	*Not at all, to a very small extent, to a small extent*	0.97
**Self-efficacy: Career advancement (4)** *Perceived ability to advance in career*	1–7 False/True	≤ 5	*Completely false, somewhat false, slightly false, neutral, slightly true*	0.83
**Self-efficacy: Research (4)** *Self-confidence in ability to be successful in research*	1–6 Confidence	≤ 3.5	*Not at all confident, slightly confident, somewhat confident*	0.87
**Self-efficacy: Work-life integration (5)** *Self-confidence in ability to manage work and personal responsibilities*	1–6 Confidence	≤ 3.5	*Not at all confident, slightly confident, somewhat confident*	0.90
**Vitality (4)** *Find work energizing and personally meaningful*	1–6 Frequency	< 5	*Never, very rarely, rarely, occasionally*	0.89
**Burnout (1)** *Frequency of feeling burnt out*	1–6 Frequency	≥ 5	*Frequently, very frequently*	--
**Relationships/inclusion/trust (5)** *Faculty relationships, feelings of inclusion and trust*	1–6 Frequency	≤ 2.5	*Never, very rarely*	0.89
**Intention to leave academic medicine (1)** *Seriously considered leaving academic medicine in last 12 months*	1–5 Agreement	≥ 4	*Somewhat agree, strongly agree*	--
**Identity self-awareness (4)** *Frequency of considering one’s identities and cultural lens*	1–6 Frequency	≤ 3.5	*Never, very rarely, rarely*	0.82
**Valuing diversity: Attitudes and recruitment (9)** *Valuing diversity in work teams, recruitment, and advancement*	1–6 Untrue/ true of what I believe	≤ 3.5	*Very untrue, mostly untrue, somewhat untrue*	0.89
**Anti-sexism & anti-racism skills (4)** *Ability to identify and respond to gender, race, and ethnicity inequity*	1–7 False/True	≤ 5	*Completely false, somewhat false, slightly false, neutral, slightly true*	0.84

The survey was distributed to all subjects in late fall 2020. Nonrespondents were sent twice-weekly email reminders with follow-ups by SMS or phone from an external survey data collection center.

### Analysis

Existing study scales were tabulated and their psychometric properties assessed by item correlation and Cronbach’s alpha (Table 1) using SAS/STAT Version 9.4 for Windows, 2006 (SAS Institute: Cary, North Carolina) [27, 28]. New survey items were reviewed in conceptual groups and examined by classical item analysis, factor analysis, and IRT modeling to arrive at a final set of three cross-cultural scales: identity self-awareness, valuing diversity (attitudes and recruitment) and anti-sexism/anti-racism skills (Table 1).

For each of the 11 domains described in Table 1, we tested subgroup differences along three dimensions: gender (women vs. men), degree (Ph.D. vs. M.D. or M.D./Ph.D.), and race and ethnicity (underrepresented in medicine by NIH criteria vs. non-underrepresented). We conducted nonparametric statistical tests (e.g., Mann-Whitney rank sum) using the full range of scale values to assess for subgroup differences. Because we conducted 33 statistical tests (11 domains × 3 dimensions, excluding regression analyses), the risk of a “false-positive” statistically significant result increases. With 33 statistical tests, we would expect up to two false-positive tests with *P* < 0.05 (i.e., 5% of 33 will have a significant *P*-value by chance alone given there is truly no difference between subgroups). However, for test results of *P* < 0.01, there is a 1% chance (less than one of the 33 tests) of a false-positive result under the assumption of no subgroup differences. Besides acknowledging the magnitude of risk of a Type 1 error (false-positive), we did not adjust for multiple comparisons.

**Figure 2.**
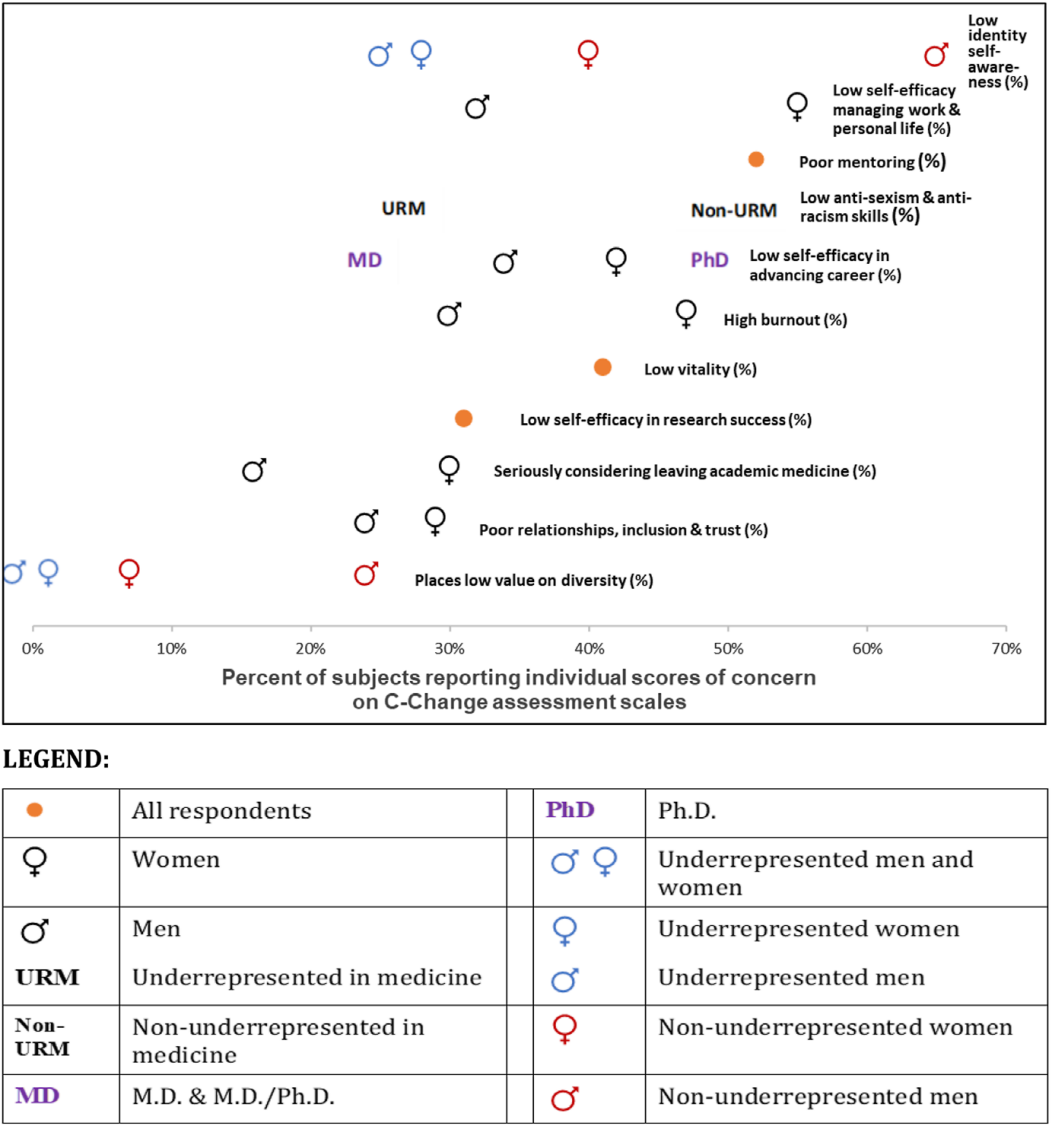
**Percent of subjects reporting individual scores of concern on C-Change assessment scales among 146 midcareer biomedical researchers completing the C-Change participant survey in fall 2020.** Non-URM and URM: non-underrepresented and underrepresented in medicine. Individuals from racial and ethnic groups that are adequately represented and have low representation, respectively, in the health-related sciences and STEM fields on a national basis, as designated by the National Institutes of Health and the National Science Foundation.

When nonparametric tests for ordinal data demonstrated a difference across subgroups (*P* < 0.05), we sought to simplify the presentation of results by dichotomizing the scale or item (Table 1 and Fig. 2). With the exception of seriously considering leaving academic medicine, the dichotomized data were used only to make comprehension easier, as statistical analyses were always performed on the full response scale.

To examine predictors of seriously considering leaving academic medicine, we dichotomized the measure into “likely” (*somewhat agree* and *strongly agree*) and “unlikely” (*strongly disagree, somewhat disagree,* and *neither agree not disagree)*, and used Poisson regression with robust standard errors (rather than logistic regression) because of the relatively high prevalence of the outcome and because the procedure provides relative risks rather than odds ratios [29].

For the three ordinal cross-cultural variables – identity self-awareness, valuing diversity, and anti-sexism/anti-racism skills – we used ordinal logistic regression with robust standard errors to assess the combined effect of gender and race and ethnicity.

All inferential statistics were estimated using Stata 14.2 or 15 (StataCorp: College Station, TX) [30].

Brandeis University Human Subjects Protection IRB approved this study (IRB #19127R-E).

## Results

Among the 148 individuals selected to participate in the study, 146 completed the baseline survey (Table 2). There were 60 subjects (41%) with an M.D. or M.D./Ph.D. degree and the remainder had Ph.D. or equivalent degree only. The eligibility criteria for research success were satisfied when the participant was the principal investigator on a K-award only (*n* = 47), an NIH R01 or R01-equivalent only (*n* = 43), both a K-award and R01 (*n* = 29), or a substantial non-NIH research grant (*n* = 27). None had two or more R01 or R01-equivalent grants, and none held the rank of instructor or professor. Participants represented 58 medical schools in 29 states. Our efforts to oversample from racial and ethnic groups considered underrepresented in medicine yielded a subgroup of 45 (31%).

**Table 2.** Characteristics of 146 midcareer biomedical researchers completing the C-Change participant survey in fall 2020

	Percent (n)
Gender	
Men	43% (63)
Women	57% (83)
Race and ethnicity	
Underrepresented in medicine*	31% (45)
Non-underrepresented in medicine*	69% (101)
Race and ethnicity by gender	
URM men	14% (20)
URM women	17% (25)
Non-URM men	29% (43)
Non-URM women	40% (58)
Degree	
Ph.D.	59% (86)
M.D.	31% (45)
M.D., Ph.D.	10% (15)
Rank	
Assistant professor	52% (76)
Associate professor	48% (70)
Research award	
NIH K-award recipients	32% (47)
NIH R01 or equivalent award recipients	29% (43)
Both NIH K and R01 awards	20% (29)
Non-NIH major award	18% (27)
Median percent time conducting research *(IQR)*	75% (50-90)
Median years since first academic appointment *(IQR)*	8 (6-11)
Median number of publications *(IQR)*	29 (18-42)

IQR, interquartile range; URM, underrepresented in medicine; Non-URM, adequately represented in medicine

* Non-underrepresented and underrepresented in medicine: Individuals from racial and ethnic groups that are adequately represented and have low representation, respectively, in the health-related sciences and STEM fields on a national basis, as designated by the National Institutes of Health and the National Science Foundation.

We have organized the presentation of results into 4 sections: (1) perceptions of mentoring; (2) self-efficacy in the domains of career advancement, research, and work-life integration; (3) attitudes and experiences at work, including intention to leave academic medicine; and (4) cross-culture issues. Within each section, we describe any differences found across the 3 dimensions of gender, race and ethnicity, and degree.

### Mentoring

Mentoring quality was poor for 76 of 145 respondents (52%; 95% CI: 44%–61%). There were no statistically significant differences in mentoring quality by gender, race and ethnicity, or degree. Among the 10 items that comprise the mentoring quality scale [31] (Table 3), the mentoring activity rated lowest was help in assessing how well your professional activities align with personal values, with 75% responding *not at all*, *to a very small extent*, or *to* a *small extent*. In comparison, 45% had poor mentoring in planning how to achieve research goals, and 56% had poor mentoring in learning the skills needed to succeed in their careers.

**Table 3.** C-Change mentoring quality assessment scale components included on the C-Change participant survey

*“In the past 12 months in your current position at your institution, to what extent has mentoring helped you …”*
formulate your career goals?
formulate your research goals?
plan how to achieve your career goals?
plan how to achieve your research goals?
learn the skills needed to succeed in your career goals?
learn the skills needed to succeed in your research goals?
find the resources you need?
have a sponsor/champion/network to advance your career or your work?
plan how to achieve your personal goals?
assess how well your professional activities align with your personal values?

### Self-efficacy in Career Advancement

Low self-efficacy in career advancement was evident for 56 of 145 (39%; 95% CI: 31%–47%). For example, one scale item asked whether respondents felt confident in their ability to overcome potential professional career barriers. Fifty percent responded that this statement was either *completely false*, *somewhat false*, *slightly false*, *neutral*, or *slightly true*. Lower career self-efficacy was more common among women than men (rank sum, *P* = 0.04), with an even larger difference between Ph.D. scientists and physician investigators (rank sum, *P* < 0.0005). Among Ph.D. scientists, 49% had low self-efficacy in career advancement, compared with 24% of physician investigators.

### Self-efficacy in Research Success

Respondents had more confidence in their research success than in career advancement. Only 45 of 146 (31%; 95% CI: 23%–39%) had low self-efficacy in research success. For example, for the item asking about confidence in securing research funding, 38% responded they were *not at all confident*, *slightly confident*, or *somewhat confident*. For this domain (unlike for self-efficacy in career advancement), there were no subgroup differences among the three groups we tested, with *P* = 0.61 for the rank sum test of the difference between Ph.D. researchers and physician investigators.

### Self-efficacy in Work-life Integration

A large percentage of respondents had low self-efficacy in their ability to successfully manage responsibilities at work and outside work: 65 of 146 (45%; 95% CI: 37%–53%). Women were less confident than men (rank sum, *P* = 0.01). For example, for the item measuring confidence in succeeding at work without sacrificing personal or family commitments, 52% of women and 29% of men responded they were *not at all confident* or *slightly confident*.

### Attitudes and Experiences at Work

#### Vitality and burnout

Low levels of vitality were reported by 60 of the 146 participants (41%; 95% CI: 33%–50%). For example, 38% of faculty reported *never* or *only occasionally* looking forward to going to work. High levels of burnout were also common: 40% (95% CI: 32%–49%) reported feeling burnt out *frequently* or *very frequently*. Although burnout and vitality were certainly associated (Spearman *r* = −0.44, *P* < 0.0005), they are not synonymous, as only 25% (36 of 146) had both low vitality and high burnout.

There were no differences in vitality by gender (rank sum, *P* = 0.12). Women, however, reported more burnout than men (rank sum, *P* = 0.01). Women were four times as likely to endorse the maximum response option for burnout (*very frequently*) compared to men (20% vs 5%).

#### Relationships, inclusion, and trust

On the scale measuring relationships with colleagues and feelings of inclusion and trust, 39 of 145 (27%; 95% CI: 20%–35%) had scores indicating poor relationships, lack of feeling included, and mistrust. Women had worse scores on this scale than men (rank sum, *P* = 0.02). For example, for one of the scale items, 35% of women reported *frequently* or *very frequently* needing to hide what they really think, compared with 22% of men.

#### Intention to leave academic medicine

Among the 146 respondents, 35 (24%; 95% CI: 17%–32%) had seriously considered leaving academic medicine within the prior 12 months. Women were more likely than men to have seriously considered leaving academic medicine (rank sum, *P* = .003). There were no significant differences by degree or by race and ethnicity.

#### Predictors of seriously considering leaving academic medicine

There was a strong dose-response relationship between amount of burnout and likelihood of seriously considering leaving academic medicine (rank sum, *P* < 0.0005), ranging from 0% among those with the lowest burnout (response options 1 or 2 on 6-point single-item response scale) to 60% among those with the highest burnout (response option 6 out of 6).

The relationship between vitality and the likelihood of seriously considering leaving academic medicine was even stronger (rank sum, *P* < 0.0005): for every one-point decrease (worse) in the six-point vitality scale, the probability of seriously considering leaving academic medicine more than doubled, ranging from 4% among those with highest vitality score (5.75–6) to 80% among those with the lowest vitality scores (1–3.25).

When considered jointly in Poisson regression modeling, both burnout and vitality were independent predictors of seriously considering leaving academic medicine, illustrating that they are related, but separate, concepts. After adjusting for burnout, those with low vitality (< 5 on vitality scale) were three times (95% CI: 1.4–6.4) as likely to seriously consider leaving academic medicine compared with those with high vitality (*P* = 0.006). And after adjusting for vitality, those with high burnout (5 or 6 on 6-point response scale) were 2.6 times (95% CI: 1.3–5.5) as likely (*P* = 0.01) to seriously consider leaving academic medicine compared to those with low burnout.

Two other variables were strong predictors of seriously considering leaving academic medicine: mentoring quality and the measure of relationships, inclusion, and trust (as single predictors, each *P* < 0.0005 in a Poisson regression). For every one-point improvement in mentoring quality score, there was a 30% (95% CI: 15%–42%) reduction in the probability of seriously considering leaving academic medicine. For every one-point improvement in the scale measuring relationships, inclusion, and trust, there was a 41% (95% CI: 24%–53%) reduction in the probability of seriously considering leaving academic medicine.

### Cross-Cultural Issues

#### Identity self-awareness

Identity self-awareness is a crucial step in minimizing sexism, racism, and marginalization, but 63 of 146 faculty (43%; 95% CI: 35%–52%) had low levels of identify self-awareness. Non-underrepresented men were much less likely to have thought about their own cultural identity compared with non-underrepresented women (ordinal regression, *P* < 0.0005) and compared with underrepresented racial and ethnic groups (*P* < 0.0005), with a significant gender by race and ethnicity interaction (*P* = 0.03). For example, 65% of non-underrepresented men had low self-awareness, compared to 40% of non-underrepresented women, 28% of underrepresented women, and 25% of underrepresented men.

#### Valuing diversity

Only 14 of 144 (10%; 95% CI: 5%–16%) respondents had scores indicating they placed a low value on diversity. Non-underrepresented men reported valuing diversity less than non-underrepresented women (ordinal regression, *P* < 0.0005), and much less than underrepresented men and women (*P* < 0.0005), with a significant gender by race and ethnicity interaction (*P* = 0.02). For example, 24% of non-underrepresented men put a low value on diversity, compared to 7% of non-underrepresented women and 0% of underrepresented men and women.

#### Anti-sexism and anti-racism skills

Sixty-three of 146 (43%; 95% CI: 35%–52%) respondents felt ineffective at identifying and responding to gender inequities and race or ethnicity inequities. Those from non-underrepresented race and ethnicity groups reported lower anti-sexism and anti-racism skills compared with those from underrepresented groups (rank sum, *P* = 0.0007). For example, 51% of respondents from non-underrepresented groups felt ill-prepared to identify and respond to sexism or racism compared to 27% of those from underrepresented groups. There were no significant gender differences (rank sum, *P* = 0.30).

## Discussion

These findings reveal that the USA may be at risk of losing already successful medical school research faculty. Our results show that this risk is related to the nonrelational culture in medical schools, inadequate mentoring, burnout, and low vitality. In presenting these results, we focus on the percent of faculty reporting unfavorable experiences that can be barriers to advancing their research careers. These areas of concern represent opportunities to sustain and vitalize promising midcareer investigators.

### Lack of High-Quality Mentoring

Although mentoring is considered a gateway to success, the literature corroborates a concerning lack of effective mentoring for research faculty [34]. Our prior research has demonstrated the association between inadequate mentoring and lower self-efficacy in career advancement; lower sense of relationships, inclusion, and trust; increased consideration of leaving one’s institution; and lack of values alignment [11,32,34]. Particularly worrisome is that Ph.D. scientists have lower self-confidence in their career advancement than physician investigators. Mentoring initiatives that include both Ph.D. and physician investigators may be one approach to mitigating this [35–37]. Training programs for mentors may help ensure the application of evidence-based practices, promote a culture of mentoring in medical schools, and help sustain a flourishing and diverse research faculty.

We found that research faculty receive mentoring that is lacking in both quantity and quality. In addition to receiving little help for achieving research goals and learning skills to succeed in one’s career, over a quarter of faculty from underrepresented groups and half of faculty from non-underrepresented groups reported that values alignment had not been addressed at all in their mentoring experience. Prior research has shown that values alignment is closely correlated with vitality [11]. Clarification of values and their alignment can shift the mentoring paradigm to focus on the mentee and necessitates welcoming the mentee’s identities and values, which may be different from their advisors and mentors.

### Sample Representativeness

Our sample has important strengths, including its national scope, comprising faculty from 58 medical schools in 29 states. The sample represents midcareer faculty with demonstrated research acumen by virtue of success in acquiring substantial extramural research funding as principal investigator.

The purpose of the sample was to conduct a randomized trial and then a longitudinal cohort study to test the effectiveness of our faculty development intervention over five years. It is impossible to know either the direction or strength of volunteer bias on any of the variables studied. Those who volunteered because they were attracted to the intervention might on average be high achievers looking to further advance their careers or they might be struggling with their career choices and hoping for a boost. Nonetheless, to our knowledge, no similar comprehensive study of midcareer investigators exists.

In our comparison of underrepresented and non-underrepresented groups across the 11 domains, there were no differences in mentoring received, vitality, burnout, trustworthy relationships among faculty, self-efficacy, or intention to leave academic medicine. One possible explanation may be the extensive cultural diversity among faculty *not* considered underrepresented by the NIH categorization, which includes immigrants and faculty of color, particularly those of historically excluded Asian and Southeast Asian groups, as well as Arab American/Middle Eastern groups. The dichotomy proposed by the NIH – based on whether the simplistic race and ethnicity characteristic in medicine is proportional to the US population – does not capture other axes of marginalization, such as inequity on the basis of national origin, skin color, gender identity, and sexual orientation, among other identities (e.g., immigrant/refugee status). The members of the non-underrepresented group comprised both historically included (White) and historically excluded (e.g., Asian, Middle Eastern) groups. We hypothesize that experiences of discrimination and intersectional identities impact faculty. Our findings highlight the need for more rigor in assessing diversity in medical school research faculty.

### Retaining Diverse Research Faculty

Our prior work showed that intention to leave academic medicine for all faculty was associated with significant ethical and moral distress, and a lower sense of relatedness and inclusion expressed as feeling isolated and invisible [9]. The current finding that nearly half of research faculty experienced conflict between their personal values and professional activities aligns with prior research [11,32]. Furthermore, our data show that the risk of losing faculty is particularly high among women. This gender gap was related to faculty dissatisfaction and not to personal or family reasons suggesting the need for further research into women’s dissatisfaction. Our findings of limited self-awareness of cultural identity and low ability to identify and respond to gender, race, and ethnicity inequity in our sample, which was particularly striking in non-underrepresented male faculty, highlight the need for activities to improve cultural awareness and anti-racism and anti-sexism skills in medical schools.

The “minority tax” [33] may be doubly levied upon faculty identifying both as women and from underrepresented groups, thus contributing to dissatisfaction and lowering vitality. This underscores the need for mentoring initiatives that recognize and seek to combat racism, sexism, and other forms of systemic injustice. Education and training are needed to ensure faculty are equipped to promote inclusion and equity, particularly in mentoring relationships. To address such issues, the NIH have initiated the Plan for Enhancing Diverse Perspectives requirement for research proposals with the goal of increasing the diversity of research teams that the NIH fund [17].

## Conclusion

Vitality, burnout, quality of mentoring, relationships, feelings of inclusion, and trust were all independently linked to the consideration of leaving academic medicine. The linkage of burnout and intention to leave academic medicine aligns with prior studies in a single academic institution [12]. Our findings strongly suggest that efforts to retain and sustain faculty – creating a setting in which they can flourish – must involve structural changes to mitigate burnout along with efforts to nurture vitality by fostering a relational culture within the workplace and effective mentoring.
